# Product development and characterization of the Ayurvedic herbo-mineral-metallic compound- *Hridayarnava Rasa*

**DOI:** 10.1016/j.jaim.2024.100886

**Published:** 2024-05-17

**Authors:** Chandrashekhar Y. Jagtap, Ashwini Kumar Mishra, Mukesh Nariya, Vinay J. Shukla, Pradeep Kumar Prajapati

**Affiliations:** a Central Ayurveda Research Institute, Jhansi, Uttar Pradesh - 284003, India; b Pharmacology Lab, ITRA, Gujarat Ayurved University, Jamnagar, Gujarat - 361008, India; c Pharmaceutical Chemistry Lab, ITRA, Gujarat Ayurved University, Jamnagar, Gujarat - 361008, India; d Dr. Sarvepalli Radhakrishnan Rajasthan Ayurved University, Jodhpur, Rajasthan - 342037, India

**Keywords:** Bhasma, Copper, *Hridayarnava Rasa*, Mercury, Standardization, *Tamra*

## Abstract

**Background:**

Herbo-mineral-metallic formulations are an inseparable part of the Ayurveda system of traditional medicine. *Hridayarnava Rasa* (HR) is a preparation containing metals like copper, sulphur, and mercury in processed forms and other herbs that do not produce toxic effects and adverse drug reactions when taken in appropriate dosage. Ayurveda practitioners use it in treating cardiac diseases like hypertension, cardiotoxicity and many more. The *rasa*-*aushadhis* possess characteristics such as rapid efficacy, little dosage required, and extensive therapeutic applicability. *Hridayarnava Rasa* [AFI Part-1, 20:55] has been employed for the treatment of various diseases from ancient times. A systematic study of these formulations manufacturing is required to maintain their quality, safety, and efficacy is a need of time to protect the immense faith of patients in Ayurveda.

**Objectives:**

The present study aimed to prepare HR as per standard operating procedures mentioned in the classical text and to characterize it physio-chemically using advanced analytical techniques.

**Materials and Methods:**

HR was prepared and physicochemical analyses and assay of elements by ICP-AES were carried out as per Ayurvedic Pharmacopoeia of India (API). Powder X-ray diffraction (XRD), Field emission gun scanning electron microscopy with energy dispersive spectroscopy (FEG SEM, EDAX), CHNS-O analysis, Fourier Transform Infrared Spectroscopy (FTIR), Thermo-gravimetric analysis (TGA), Particle size distribution analysis (PSD) was carried out.

**Results:**

The XRD analysis of HR showed the presence of unreacted sulphur and sulfides of copper and mercury. FEG SEM revealed the particles in the form of aggregates as nanocrystallites in the range of 100–1000 nm. Elemental analysis showed the presence of copper, sulphur, and mercury in major, along with traces of iron, calcium, sodium, potassium, and magnesium. In FTIR analysis, 18 peaks were observed, which strongly suggests the presence of various organic groups. In the TGA, four peaks were seen, which can be attributed to sulphur volatilization and oxidative changes in mercury. In PSD analysis, 50% of the material was found below 16.40 μm.

**Conclusion:**

To establish a piece of fundamental knowledge and ensure uniformity of these *rasa-aushadhis*, it is imperative to conduct an analysis of their characteristics as per classical texts and modern analytical techniques. Additionally, it is crucial to investigate the significance of each procedural step included in the preparation process. The inferences drawn are helpful as an essential aid for quality assurance and standardization of this herbo-mineral-metallic formulation.

## 1 Introduction

Rasa Shastra, a distinct field within the realm of Ayurveda, focuses on the study and application of substances referred to as ‘Rasa dravyas’, with the term itself translating to ‘Science of Mercury’. The term “rasa” mostly refers to Parada, which is a Sanskrit word for mercury. The term “*Rasa-aushadhis*” refers to preparations that are composed of mercury, burned metals, and minerals. These formulations are classified as herbo-mineral-metallic preparations [1]. Metal-based nanomaterials can be created using a variety of methods, including microwave hydrothermal, flash combustion, co-precipitation, sol-gel, and citrate gel procedures [2,3]. The metal-based nanomaterials have increased tremendously in recent decades as a result of the discovery of several particularly relevant iron-containing polycrystalline materials..

By understanding the structural, optical, and magnetic properties of these nanomaterials, targeted medication administration can be achieved by delivering metal-based nanomaterials to the specific tissues is a valuable site-directed application in the field of nanotechnology [4]. In Ayurveda, the metal-based formulations are the *rasa-aushadhis,* which possess properties such as rapid efficacy, minimal dosage required, and high medicinal value. There are four distinct procedures for the creation of these formulations, namely *Khalviya Rasayana, Parpati Rasayana, Kupipakawa Rasyana,* and *Pottali Rasayana*. The *Hridayarnava Rasa* (HR) is a type of *Khalviya Rasayana*, which is a herbo-mineral-metallic preparation [5,6]. For management of cardiovascular diseases (CVDs) some of ayurvedic herbal and herbo-mineral-metallic formulations utilized are *Mahamrutyunjaya rasa, Pushkaramooladhya Churnam, Hridayarnava Rasa, Dasamoolahareetaki, Sankara vati, Brahma Rasayan, Heerak Bhasma, Punarnava Baladi Kashayam, Arjuna ksheerpakam* etc. The science of Ayurveda is rich in metal, herbal, and mineral based formulations as compared to other medicinal systems. Extensive research on the safety aspects of such Herbo-mineral-metallic formulations is necessary to enhance their acceptance among patients as well as modern medical practitioners. These formulations play a vital role where the therapeutic gap is noticed in the areas of chronic diseases like cancer and CVDs [7]. The data generated by adopting the scientific methodology to standardize and characterize Ayurvedic medicines will help in their global acceptance.

While fixing the standards of excellence for physicians, paramedical workers and drugs, the Ayurveda has been conscious to maintain standards for quality and treatment schedule of Ayurvedic formulations. *Hridayarnava Rasa* (HR) is an essential herbo-mineral-metallic drug that is extensively used in clinical conditions like hypertension, ischemic heart disease, hyperlipidaemia, inflammatory diseases, diabetes, and respiratory disorders [8]. Traditionally, the process to be followed for manufacturing a drug and some parameters for characterization to assess its quality have been mentioned in Ayurvedic texts, some of which are parameters like *Rekhapurnatva, Varitaratva, Apunarbhava,* etc, are mentioned for *Bhasma* [9]. There is a lack of advanced analytical standards for the characterization of HR, as the traditional method may not result in producing high-quality products and batch-to-batch reproducibility. So, standardization is needed for these types of herbo-mineral-metallic formulations with advanced analytical tools such as X-ray diffraction (XRD), Atomic Emission Spectroscopy with Inductively Coupled Plasma (AES ICP), Fourier Transform Infrared Spectroscopy (FTIR), CHNS-O analysis, Thermo-Gravimetric analysis (TGA), Field emission gun Scanning Electron microscopy with Energy dispersive spectroscopy (FEG-SEM, EDAX) and Particle size distribution analysis (PSD). Since *Rasashastra* has chemistry as its closest in order to understand its principles and to evaluate them, to establish scientific reasoning, it is required to use today's available technologies for these processes and parameters. Along with this, for the specification and standardization of any substance, today, the whole world is following standard analytical parameters of modern sciences. An analytical study of a drug also helps to understand its pharmacokinetics and pharmacodynamics. Assessment of drugs at their various levels not only ensures quality of product but also indicates the genuine quality of raw materials. Therefore, the analytical study serves a dual purpose of scientifically understanding the concepts of *Rasashastra* as well as evaluating them for efficacy, quality, purity, and strength. A chief objective of the analytical study in the present context is to know the particular chemical configuration and the physio-chemical changes that occur during and after different pharmaceutical procedures like *Shodhana, Bhavana, Marana,* etc. The technique of Bhavana is a pharmaceutical procedure commonly used in Ayurveda with various implications for pharmacology and therapeutics. This approach is utilized to generate numerous traditional herbal or Herbo-mineral-metallic formulations that are widely recognised and utilized. The *Bhavana* method is claimed to enhance the efficacy of pharmacological action, potentially leading to a decrease in the required therapeutic dosage [10]. In a nutshell, thanks to scientific progress and technological refinement, we now have access to a standardized database of medicinal agents derived from botanical, inorganic, herbo-mineral, metallic, aquatic, or animal origin, as well as a wide range of finalized preparations and formulations. This will give a scientific basis for Ayurvedic pharmaceuticals and aid in the globalization of Ayurveda. This research set out to analytically standardize HR formulation those made with *Shuddha Parada* (processed Mercury), *Shuddha Gandhaka* (purified sulphur), *Tamra Bhasma* (Copper microparticles), Haritaki (*Terminalia chebula* Retz.), Bibhitaki (*Terminalia bellirica* Gaertn. Roxb.), Amalaki (*Phyllanthus emblica* L.) and kakamachi drava (juice of *Solanum nigrum* L.).

## 2 Material and methods

All the materials were purchased from the local market of Jamnagar, Gujrat, India, and authenticated by Rasa Shastra experts. The Herbo-mineral-metallic formulation was prepared in the test center of *Rasashastra* and *Bhaishajya Kalpana*, Institute for Post Graduate Teaching and Research in Ayurveda (I.P.G.T. & R.A.), Gujarat Ayurved University, Jamnagar, Gujarat, India.

### 2.1 Standard procedure for the preparation of *Hridayarnava Rasa*

#### 2.1.1 *Parada Shodhana* (purification of mercury)

1000 g garlic cloves were removed from garlic bulbs. These garlic cloves were 800 g in weight which were subjected to washing and then peeled off. 800 g of mercury, garlic bulbs, and 380 g of rock salt were taken into a mortar pestle for trituration for 10 h for 3 days. The colour variations were documented. The mercury was cleaned with lukewarm water at 50 °C. Mercury (Hg) was kept in a clear glass container. The ICP-AES (Spectro Analytical Instruments GmbH, Germany; Model: ARCOS, Simultaneous ICP Spectrometer) analysis was performed by taking 5 g of material from the first batch [11,12].

#### 2.1.2 *Gandhaka Shodhana* (purification of sulphur)

350 g of sulphur was collected, weighed, and crushed in a stone mortar. A jar with enough milk (700 ml) to cover all of the sulphur was taken, and its mouth was wrapped with thin white fabric and fastened. An equal amount of ghee was poured into an iron pan and cooked over a low flame. When the ghee melted, powdered sulphur was added, and the sulphur was melted before being put through a cloth into a receptacle holding milk to filter the impurities present in the sulphur. The obtained sulphur was then washed with hot water to remove the ghee. The final amount of obtained purified sulphur was found to be 345 g [13].

#### 2.1.3 Preparation of *kajjali* (black sulphide of mercury)

500 g of purified mercury and 500 g of purified sulphur were triturated together in a mortar for 36 h. The *kajjali* of fine quality weighing 990 g was obtained [14].

#### 2.1.4 *Samanya Shodhana* of *Tamra* (simple purification of copper)

It was accomplished by an ancient ayurvedic process known as the *Nirvapa* technique (heating and quenching) in the media given in the following order*: Tila Taila* (Sesame oil)*, Takra* (buttermilk)*, Gomutra* (Cow urine)*, Aranala* (sour gruel), and *Kulattha Kwatha* (decoction of *Dolichos biflorus L.* seeds). The procedure was carried out seven times in each medium. Equipment such as a gas burner, an iron pan, an iron ladle, an iron rod, two stainless steel pots, a spatula, a measuring cup, a weighing machine, a pyrometer, and a thermometer that measured temperatures up to 360 °C were employed for this purpose. Further, in brief, the raw *Tamra* (raw copper) flakes were placed in an iron pan, roasted on a gas burner until they reached a red-hot temperature, and then quenched in a certain liquid medium that was placed in a stainless-steel container. After it had cooled, *Tamra* was removed from the jar and placed again in the iron pan, where it was heated and then quenched. The procedure was carried out a total of seven times with each medium. On each and every occasion, the exact identical quantity of new media was considered gravimetrically. The temperature of the iron pan and *Tamra*, when they were in a condition of red hot, was recorded [15].

#### 2.1.5 *Vishesha Shodhana* of *Tamra* (distinctive purification of copper)

*Samanya Shodhita Tamra* was stored in a *pottali* (small muslin cloth bag) that had been woven out of cotton fabric. An iron rod was inserted through the *muslin cloth bag's* apex, after which it was hung in a steel jar, and 3 L of *cow urine* were poured to ensure that it was entirely submerged. The *Swedana* (boiling) procedure was carried out for a total of 3 h. To ensure that there was still enough *Cow urine* to dip the *muslin cloth bag*, additional *Cow urine* was added. After waiting for 3 h, the *muslin cloth bag* was removed, and *Tamra* was washed in warm water and then dried. The temperature of the *Cow urine* was monitored and kept constant during the whole operation [16].

#### 2.1.6 Preparation of *Tamra Bhasma* (copper microparticles formed by incineration)

*Tamra Bhasma* is a metallic formulation used in traditional Indian medicine. It is made by purifying copper (*Shodhita Tamra*) and then heating it to high temperatures in a controlled environment. The resulting ash is then processed to create a fine powder of copper microparticles. In the present study, for the preparation of *Tamra Bhasma* using the classical method mentioned in chapter 5 and verse number 53 of the ancient text named Rasaratna-Samuucchaya was followed. In brief, an equal quantity of *kajjali*, *Shodhita Tamra* and *nimbu swaras* (Juice obtained from fruits of *Citrus medica* L.) were subjected to trituration and small pellets were prepared, which were then subjected to three *Putas* (incineration cycles) in an electrical muffle furnace. The temperature conditions used for the first, second and third *puta* were 750 °C for 25 min, 650 °C for 30 min and 550 °C for 35 min, respectively. The prepared *Tamra Bhasma* was subjected to undergo through a process known as *Amritikaran*. It is very specific to particularly some specific mineral-originated raw drugs by which the remaining unwanted impurities and by-products were removed. In *Amritikaran* process, by providing the *Bhavana* (wet trituration) of *Nimbu Swarasa*, *Tamra Bhasma* was ground into a paste along with one-half of a portion of *Shuddha Gandhaka* (purified sulphur). Following the completion of the correct trituration, a spherical bolus was formed and allowed to dry in the shade. A *Surana Kanda* (tubers of *Amorphophallus campanulatus* Roxb.) weighing 2.5 kg was divided horizontally into two pieces after being cut in half. A circular depression was cut out of the centre of each of the parts. The dried bolus was maintained inside of it, and the two halves were put together to make the whole. It was then covered with a substantial quantity of purified clay-smeared muslin cloth. After that, it was dried out in the sunlight in glass glass-covered chamber to avoid environmental contamination. Following the removal of the excess moisture, the bolus was subjected to electrical muffle furnace treatment for 40 min at a temperature of 550 °C. The bolus that was found within was removed, self-cooled, ground up and then placed in an airtight non-reactive glass bottle for further classical and advanced characterization [14].

#### 2.1.7 Preparation of *Triphala Kwatha*

The weighed quantity (2 kg) of powdered fruit blend (fruits rinds of *Emblica officinalis* Gaertn., *Terminalia chebula* Retz. Fruit, *Terminalia bellirica* Roxb.) was heated at a constant temperature in the extraction vessel (in 16 L of distilled water), which ranged from 80 °C to 90 °C, for 4 h. The volume was reduced to ¼^th^ of its initial volume. The extract was filtered using a muslin cloth with a 100 m pores size. The amount of finally prepared *Kwatha* was found to be 4 L. The filtrate (*Triphala Kwatha*) was subsequently utilized in the preparation of HR [17].

#### 2.1.8 Preparation of *Hridayarnava* R*asa* (HR)

The preparation of HR was done by using the classical method mentioned in the ancient text of Rasendrasarasamgraha (also given in Ayurvedic Formulary of India Part-1, 20:55). Weighed amount (in a ratio of 1:1) of *Kajjali* and *Tamra Bhasma* were measured and subjected to trituration in porcelain mortar. One Bhavana (a cycle of wet trituration for about 4–6 h) of *Triphala Kwatha* was given after each Bhavana of *Kakamachi Swarasa* (expressed whole plant juice of *Solanum nigrum* L.). The final product was dried at room temperature and kept in air-tight glass bottle for further analysis. [Voucher numbers of herbs used in the preparation of formulation: *Kakmachi* (*Solanum nigrum* L.): IPGT&RA/PHM 6217; *Nimbu* (*Citrus medica* Watt.): IPGT&RA/PHM 1582; *Surana* (*Amorphophyllus campanulatus* Roxb.): IPGT&RA/PHM 1359; *Kulattha* (*Dolichos biflorus* L.): IPGT&RA/PHM 6894] [18].

### 2.2 Organoleptic evaluation by ancient ayurvedic methods

Samples of *Tamra Bhasma*, *Kajjali* and HR were analyzed by organoleptic parameters like colour, touch, taste, and smell. Classical parameters applied to the *Tamra Bhasma* were *Varitaratwa, Uttama/Unama, Rekhapurnatwa, Niruttha, Apunarbhavatwa, Dadhi Pariksha, Avami and Niswaduta,* which are mentioned in ancient texts of Ayurveda [[19], [20], [21], [22]] are as follows:

#### 2.2.1 *Rekhapurnata* test

A pinch of *Bhasma* was rubbed among the finger and thumb and observed if it fills the creases of fingers [23].

#### 2.2.2 *Varitaratwa* test

A small quantity of the prepared *Bhasma* powder was sprinkled over the surface of still water in a 100 ml beaker. Upon observation, it was noted that the *Bhasma* particles were floating on the surface of the water [24].

#### 2.2.3 *Unam* test

The process involves gently placing the *Bhasma* over water. If it remains afloat even when covered in grains, it is considered an *Unama Bhasma* or *Uttama Bhasma* [25].

#### 2.2.4 *Nishchandrata* test

To determine the presence of any lustrous residues or particles remaining in *Bhasma*, a small amount of *Bhasma* samples was rubbed in between the finger and thumb and observed under sunlight [26].

#### 2.2.5 *Apunarbhavata* test

The components of *Mitra Panchaka* [*Guda* (Jaggery), *Gunja* (seeds of *Abrus precatorius* L.) *Ghrita* (clarified butter)*, Madhu* (honey)*,* and *Tankana* (borax)] were combined with 10 g of *Bhasma* and pounded into pellets before being stored in *Sharava Samputa* (two earthen pots joined together). The pellets were then collected, triturated in a *Khalva Yantra* (porcelain mortar pestle), and examined for any lustred particles or gathered masses after being exposed to the same grade of heat used in the manufacture of *Bhasma* (500 °C as the maximum temperature for a duration of 30 min). The absence of metallic particles during trituration is indicative of *Bhasma* passing the *Apunarbhava* test [27].

#### 2.2.6 *Niruttha* test

10 g of *Bhasma* was measured and subjected to *Sharava* (earthen pot), and a silver leaf weighing about 0.4 g was kept inside the *Bhasma* and covered well. Another *Sharava* was placed on it, and the junction was sealed by mud-smeared clothes. After complete dryness, it was subjected to a similar grade of heat (500 °C at the highest temperature for the duration of 30 min at the highest temperature) for the preparation of *Bhasma* and after self-cooling, the silver leaf was collected and weighed. Here no change in the weight of the silver leaf suggests that *Bhasma* passes the *Niruttha* test [28].

#### 2.2.7 *Dadhi Pariksha* (curd test)

The curd was placed in a small pot, and a pinch of sample was dusted on top. There was no discolouration in the curd after 24 h. Therefore, *Bhasma* appears to pass the curd test [21].

### 2.3 Physiochemical parameters of *Hridayarnava Rasa*

The standard procedures mentioned in Ayurvedic Pharmacopoeia of India (API) were followed for testing of raw samples (*Tamra Bhasma, Kajjali, Triphala Kwatha* and *Kakmachi Swarasa*), and the prepared formulation (HR) were subjected to determination of pH, specific gravity, loss on drying, ash value, acid insoluble ash, total solid contents, water-soluble extractives, alcohol soluble extractives, mercury in raw and *Shodhita Parada* (processed or purified form), sulphur in raw and *Shodhita Gandhaka* (processed or purified form) [29,30].

## 3 Characterization

### 3.1 XRD analysis of the prepared herbo-mineral-metallic formulation

XRD crystallography was utilized in order to examine and characterize the structure of the prepared HR microparticles. In order to identify the phases of these microparticles, XRD was utilized. On a Rigaku Geiger flex diffractometer Japan, the XRD graphs of the microparticles in their as-extruded state were evaluated with Cu Kα radiation at 30 kV and 30 mA with λ = 1.54178A°. Tests were carried out at a scan rate of 5⁰/min across a range of 2θ, which extended from 15 to 75⁰ [31,32].

### 3.2 SEM-EDS studies of *Hridayarnava Rasa*

To examine the microstructure of HR microparticles as well as their crystalline shape, SEM (Scanning electron microscopy with Energy-dispersive X-ray spectroscopy) was utilized. The JEOL JSM-5510LV instrument was used to acquire the SEM pictures, and the acceleration voltage was set at 30 kV throughout the process. In order to obtain the SEM specimens for the examination of the microstructure, the strips of the microparticles were first cooled in liquid nitrogen for around 20 min, and then they were broken up into pieces. To study the crystalline morphology, the specimens prepared were then cut down into strips of microparticles with base resins into appropriately sized pieces and then etched for about an hour with a solution of potassium permanganate in a combination of sulfuric acid and orthophosphoric acid, as described in the previous works [33,34]. Before being examined by SEM, all these samples were plated with gold. EDS was utilized to confirm that the microparticles seen in the HR microstructure are copper microparticles, and SEM-EDS Cu-mapping was performed to assess the HR microparticle distribution throughout the matrix.

### 3.3 TGA, DSC and FTIR studies

The TGA (Thermo-Gravimetric analysis) experiment was conducted in a nitrogen environment with a flow rate of 50 mL/min. The temperature was gradually increased from 25 °C to 600 °C, with a heating rate of 10 °C/min. In order to assess the oxidation behaviour of the HR particles, TGA (PerkinElmer, USA; Model- Diamond TG/DTA) was also carried out under the same conditions but without the injection of nitrogen [35]. The Fourier-transform infrared (FTIR) spectroscopy was performed to obtain spectral curves of prepared HR microparticles between 7500 and 370 cm^−1^ in transmittance mode by using a Bruker Tensor 27 IR, Bruker, Germany, in order to examine specific interaction between the formulation ingredients [36,37]. DSC stands for “differential scanning calorimetry.” The DSC (DSC7020, Hitachi, Japan) method was used to investigate the thermal characteristics. The samples, each weighing 2 mg, were put in aluminum pans, and subjected to a heating rate of 10 °C/min, with the purging of nitrogen at a rate of 50–500 ml/min as they were brought from 25 °C to 725 °C.

### 3.4 Particle size distribution analysis (PSD)

The purpose of the particle size analysis was to investigate the typical dimensions of the HR microparticles as well as the particle size distribution of the prepared formulation. During the course of the research, a Laser Particle Size Analyzer Symantec Helos BF with a range of 0.1–875 μm was utilized. To prepare the samples, the samples were first dispersed in deionized water, and then the mixture was sonicated for 5 min [38].

### 3.5 ICP-AES and CHNS-O analysis

An advanced tool, an ARCOS, Simultaneous ICP Spectrometer inductively coupled plasma atomic emission spectroscopy (ICP-AES) of analysis, was employed to simultaneously detect the amount of trace elements present in prepared samples of HR. Elemental analysis was performed by a Flash smart V CHNS/O (Thermo-Fisher Scientific, Waltham, Massachusetts) and were supported by Sophisticated Analytical Instrument Facility (SAIF), Indian Institute Technology Bombay, Mumbai [39].

## 4 Results and discussion

### 4.1 Preparation of *Hridayarnava Rasa* and organoleptic evaluation by ancient ayurvedic methods and physiochemical parameters

Raw *Tamra*, *Parada* and *Gandhaka* taken for the study were analyzed for their purity. Raw *Tamra* was 99.8% pure, and its purity decreased after the *Samanya* and *Vishesha Shodhana* procedures (Table 1 in Supplementary data). This is because of the increased conversion of copper into cupric oxide (CuO) during the heating process, which gets washed away with *Shodhana* media during the washing process. The increased percentage of iron after the *Samanya Shodhana* procedure was due to the usage of an iron pan for this process. The decrease in the amount of total sulphur after *Gandhaka Shodhana* (Table 2 in supplementary data) and total mercury after *Parada Shodhana* may be due to the process of segregating sulphur from its chemical and physical impurities, as well as the removal of additional volatile contaminants that were chemically bound to the sulphur samples [40]. The changes in different liquid media used in the analysis (*Sesame oil, Takra, Cow urine, Kanji, Kulattha Kwatha*) before and after *Samanya Shodhana* of *Tamra* were reported (Table 3 in supplementary data). The ancient organoleptic evaluation results by ancient ayurvedic Methods of *Tamra Bhasma* prepared from *Shuddha Tamra* were shown in Table 4 of Supplementary data. During the preparation of *Tamra Bhasma*, *Dadhi Pariksha* (curd test) shall be considered more useful due to the fact that it is the simplest chemical test for determining whether a medicinal agent has been converted to a stable and nontoxic form [41]. Since the significance assigned to this evaluation while completing the formulation process of *Bhasma*, when *Apakwa* (improper/crude/in-process) *Bhasma* is tasted, it produces extreme salivation, dizziness, metallic taste, and pain in the head. Therefore, it is not recommended to examine the *Bhasma*'s quality by tasting it after each *Puta*. Although *Bhasma* which is black in color, is thought to be *Awami* (non-vomiting), this may not always be the case. Instead, the *Tamra Bhasma* is finished with a *Dadhi Pariksha*, a simple, safe, and chemical testing conducted within a scientific laboratory with established standards.

**Table 1:** Physiochemical parameters of *Hridayarnava Rasa* ingredients.

Parameter	*Tamra Bhasma*	*Samaguna Kajjali*	*Triphala Kwatha*	*Kakmachi Swarasa*	*Hridayarnava Rasa*
Loss on drying (% w/w)	0.95 ± 0.02	0.41 ± 0.04	–	–	0.92 ± 0.05
Ash value (% w/w)	96.98 ± 1.22	0.85 ± 0.06	–	–	39.45 ± 2.34
Acid insoluble ash (% w/w)	2.24 ± 0.42	98.02 ± 3.22	–	–	2.90 ± 0.62
Water soluble extractive (%w/w)	0.81 ± 0.12	0.31 ± 0.04	–	–	9.22 ± 0.51
Alcohol soluble extractive (%w/w)	0.92 ± 0.03	0.24 ± 0.01	–	–	4.67 ± 0.81
Total solid contents (%w/v)	–	–	15.2 ± 0.54	16.3 ± 0.36	–
Specific gravity (%w/v)	–	–	1.03 ± 0.02	1.82 ± 0.13	–
pH (10% Solution)	6.2	6.1	6.4	6.8	6.3

**Table 2:** Organoleptic characters of *Hridayarnava Rasa* ingredients.

Parameters	*Tamra Bhasma*	*Samaguna Kajjali*	*Triphala Kwatha*	*Kakmachi Swarasa*	*Hridayarnava Rasa*
Color	Black	Black	Dark Brown	Dark Green	Black
Touch	Soft, smooth	Soft, smooth	–	–	Soft, Smooth
Taste	Tasteless	Tasteless	Astringent	Bitter	Astringent
Odor	Not specific	Not specific	Characteristic	Characteristic	Not specific

**Table 3:** 2θ values of identified peaks of elements found and their crystalline phase in XRD of *Tamra Bhasma*, *Samaguna Kajjali* and *Hridayarnava Rasa* microparticles.

Tamra Bhasma		Samaguna Kajjali		Hridayarnava Rasa	
2θ value	Phase of compound	2θ value	Phase of compound	2θ value	Phase of compound
10.8004	Covelline (002) and Covellite (002) like CuS	23.1220	Sulphur	23.0197	Elemental Sulphur
23.9677	Elemental Sulphur	25.8818	Sulphur	24.6657	HgS
27.0596	Covelline (110) like CuS	26.4312	Hexagonal	26.3692	HgS
27.6639	Covelline (101) like CuS	27.7391	Sulphur	27.8566	Covelline (101) like CuS
29.2658	Cu_2_O	28.7056	Sulphur	33.3385	Covelline (110) like CuS
31.7718	Covelline (103) like CuS	30.5709	Cubic	35.2590	Covellite (112) like CuS
32.8194	Mixture of Covelline and covellite like CuS	37.0760	Hexagonal	42.3693	Cu_2_O
42.3928	Covelline (106) and Cu_2_O	43.8161	Cubic	43.6900	HgS (110) and CuS
47.9378	Covelline (110) like CuS	45.8950	Hexagonal	47.7885	HgS
48.0913	Covellite (021) like CuS	51.8058	Hexagonal Cubic	51.7978	HgS
52.7038	Covelline (108) like CuS	70.0934	Cubic	54.9515	HgS

**Table 4:** EDAX showing quantity of different elements present in *Hridayarnava Rasa* sample.

Element	k Ratio	Weight%	Weight% [Sigma]	Intensity [Corrn.]	Atomic%
Pb La	0.24415	0.193	0.310	0.9033	0.48
Hg La	0.42542	26.096	3.652	0.9362	12.201
Cu Ka	0.12975	55.708	1.362	1.1754	53.55
Fe Ka	0.00275	0.869	0.171	1.2600	0.182
Ca Ka	0.00625	0.54	0.078	0.9501	1.028
K Ka	0.01399	0.878	0.103	0.9000	1.021
S Ka	0.13459	15.527	5.811	1.0514	31.014
Mg Ka	0.00102	0.189	0.044	0.7500	0.524
**Totals**		**100.000**			

Average values of physicochemical parameters of ingredients of HR (Table 1) and organoleptic characters (Table 2) are in conformance with some of the prior investigations. A low loss on drying value indicates that moisture is nearly non-existent across all samples. The significantly elevated ash value of *Tamra Bhasma* indicates that it contains a significant quantity of inorganic substances. Smaller acid insoluble ash value indicates more *Bhasma* physiological availability. Water soluble extractive value and methanol soluble extractive values were found more in HR because it is treated with *Triphala Kwatha* and *Kakmachi Swarasa*.

### 4.2 X-ray diffraction (XRD) studies

**Fig. 1:**
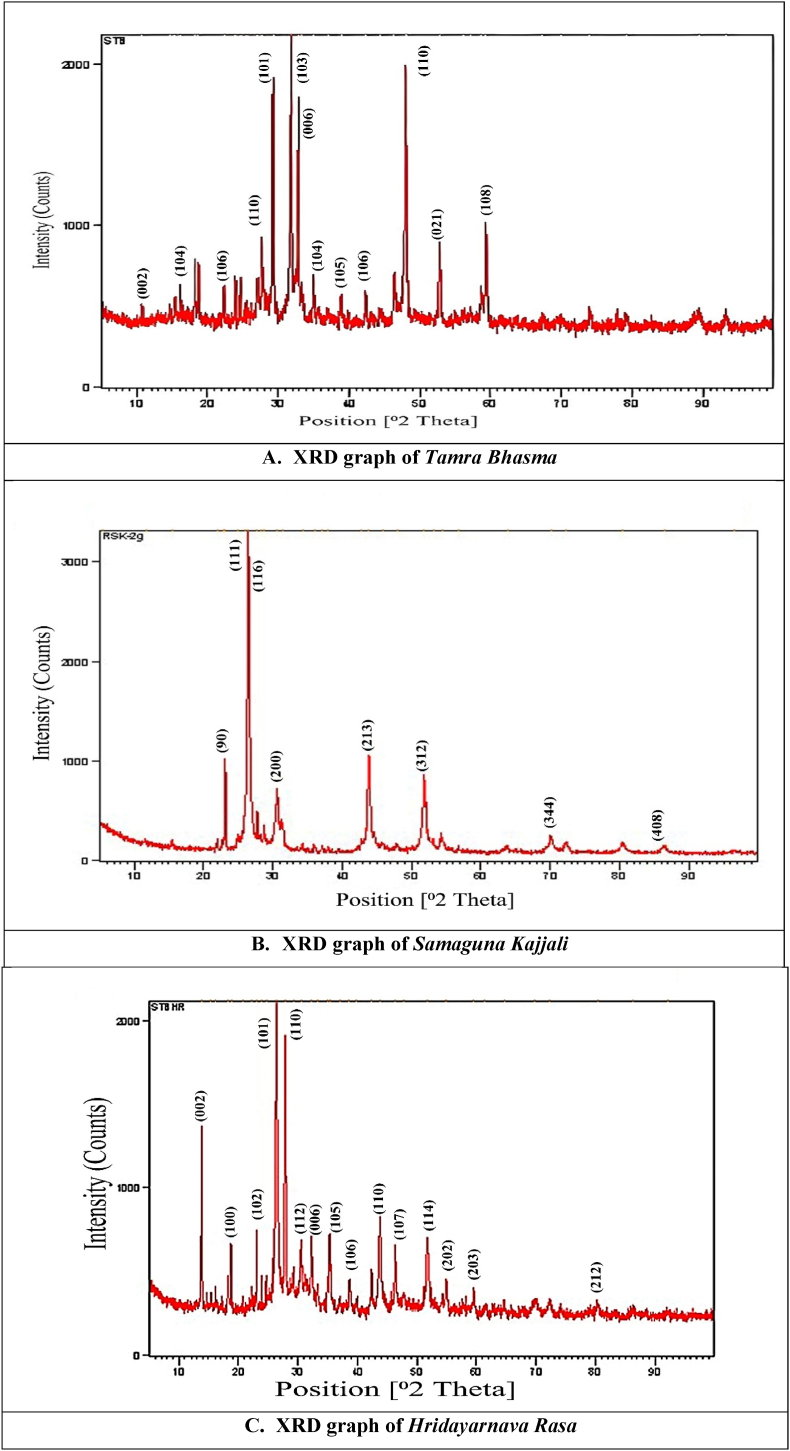
The XRD graphs show the crystallinity of prepared (A) *Tamra Bhasma* (B) *Samaguna Kajjali* (C) *Hridayarnava Rasa* (HR) formulation.

XRD of *Tamra Bhasma*, *Kajjali* and HR was carried out by comparing it with the Joint Committee on Powder Diffraction Standards (JCPDS) cards for copper sulfide-CuS (covelline and covellite phase) with numbers 85–0620 and 78–2122 along with the JCPDS cards with number 80–2192 for black sulphide of mercury (HgS). 2θ values of identified peaks compared with standard and identified crystal structure and chemical nature of these samples are depicted in Table 3. XRD analysis of *Tamra Bhasma* confirmed that the incineration treatment of copper with sulphur resulted in the formation of a mixture of phases of CuS along with the elemental sulphur in it. Some peaks of Cu_2_O were also found in it. The formation of sulphides of copper in accompaniment with sulphur is discussed earlier, and the results follow some of the previous studies [21,42]. XRD of *Samaguna Kajjali* showed unreacted sulphur along with hexagonal and cubic phases of HgS. HR also contains unreacted sulphur along with the covelline and covellite like copper sulphide and metacinnabar phase of mercury sulphide. HR is prepared by mixing *Tamra Bhasma* and *Samaguna Kajjali* in equal parts and levitated with herbal juices. Thus, in its XRD analysis, components mixed with each other are observed since no chemical reaction was expected at this step. Crystalline nature of all the samples is clear by sharp peaks exhibited in XRD graphs (Fig. 1).

### 4.3 FEG SEM analysis of *Hridayarnava Rasa*

**Fig. 2:**
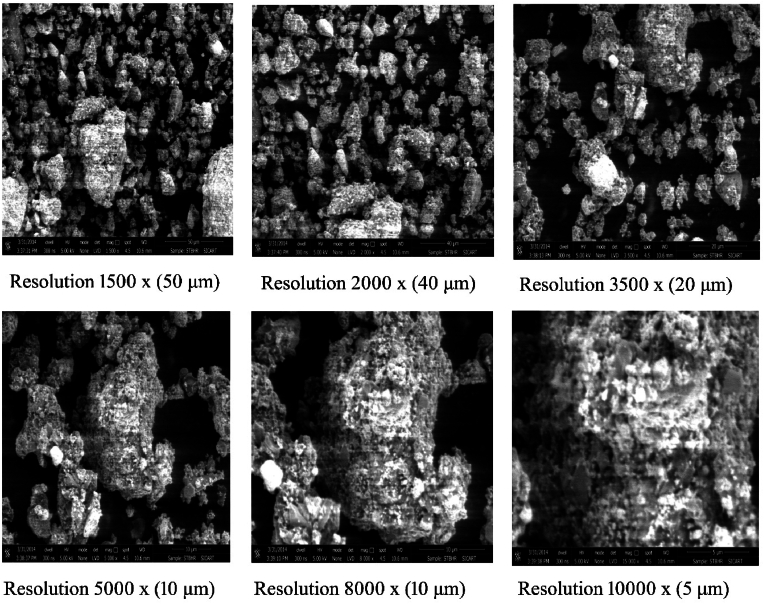
The SEM pictures displays the morphological properties of prepared *Hridayarnava Rasa* at different resolutions.

Field Emission Gun – Scanning Electron Microscope (FEG-SEM) reveals that the gross particle size of HR varies roughly from 100 to 1000 nm, and the particles exist in the form of aggregates which, in fact, does not present the actual size of an individual particle (Fig. 2). The existence of embedded smaller particles in a lump of micron sized assembly of particles was detected in the inset. The sample has dispersed fine nanoparticles of size less than ∼20 nm at its surfaces. The presence of irregular particle size in the submicron range is due to the levigation of the materials with herbal juices, which induce an organic nature to the materials. *Bhavana*, alternatively referred to as Samskara in the Sanskrit language, denotes the process of transforming the innate characteristics of a substance, resulting in the acquisition of new properties or qualitative enhancement [43]. The process of repeated grinding cycles during *Bhavana* has been observed to have potential effects on size reduction. These effects may have implications for the extraction of chemical components from the drug as well as the absorption of its elements inside the gastrointestinal tract, particularly when delivered orally. Factors such as the specific site, percentage, and extent of absorption and metabolism are likely to be influenced by this grinding process. The phenomenon of size reduction in *Bhavana* can be elucidated by the use of the “Griffith theory.” This theory posits that the presence of structural weaknesses, or defects, within all solids might potentially give rise to the formation of microscopic cracks when subjected to stress or strain, as experienced during the *Bhavana* process. According to the “Attrition theory,” the process of rubbing materials between liquid media and the surfaces of a pestle and mortar leads to Particle Size Reduction [44,45]. So, it may be due to the Bhavana procedure, nano-size crystallites get agglomerated and give rise to the micro-sized particles. Thus, it can be confirmed that HR contains nano-crystallites with submicron-sized particles, and the present study is in congruent to previous studies done by various research groups [46,47].

### 4.4 EDAX and ICP AES analysis of *Hridayarnava Rasa*

**Fig. 3:**
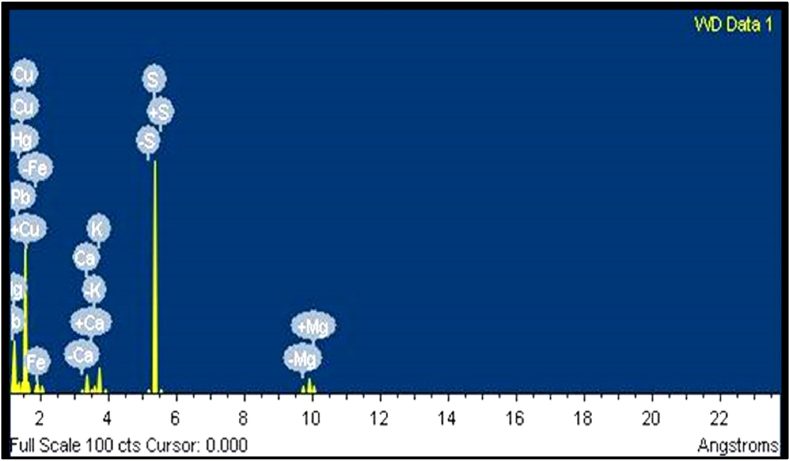
EDAX graph of prepared *Hridayarnava Rasa*.

Along with the major elements like mercury, sulphur and copper, other elements are also predictable in the final product which introduced in it during its pharmaceutical processing. EDAX is performed for the quantitative determination of bulk elemental composition whereas the ICP AES method is used to detect the elements present in traces [48,49]. Different elements were present in HR as observed in EDAX analysis (Fig. 3, Table 4). The lack of adverse effects in terms of stomach lesions can be attributed to the availability of iron (Fe), calcium (Ca), potassium (K), and magnesium (Mg), the four minerals most crucial to the proper functioning of metabolic process in body. Normal balance of fluids between internal and external cell environment is maintained by the electrolytic elements sodium (Na) and potassium (K), which are additionally present in trace amounts. Different pharmaceutical processing like *Shodhana, Marana* and *Bhavana* applied during its preparation are the possible sources of these elements. All these components, culled from many sources, work together as a cohesive whole to boost the drug's efficacy, and provide what appears to be a necessary complement in curing disorders. ICP AES analysis also showed the presence of copper, mercury, and sulphur in major proportion (Table 5). Heavy metals like arsenic, lead and cadmium were within the limit of safety recommended by World Health Organization (WHO) [48,50]. Mercury was found to be high enough but it is in sulphide form which is least toxic shown in previous studies [[51], [52], [53], [54]].

**Table 5:** *Hridayarnava Rasa* sample elemental content determined through ICP-AES analysis.

Copper	Sulphur	Mercury	Arsenic	Lead	Cadmium	Iron	Magnesium
ppm	ppm	Ppm	ppm	ppm	ppm	ppm	ppm
236.848	76.956	236.306	1.916	0.35	ND	4.025	1.075

**ND** – Not detected (less than 0.01 ppm).

### 4.5 TGA, DSC and FTIR

**Fig. 4:**
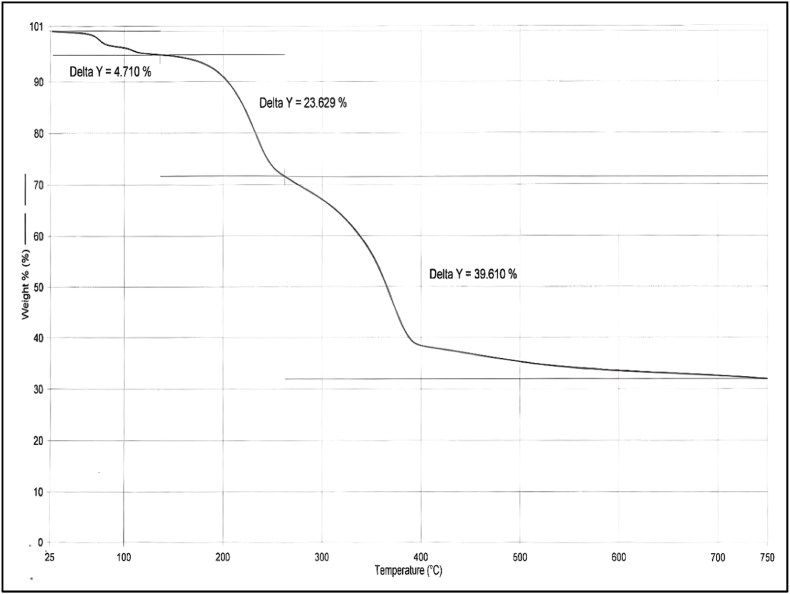
TGA analysis of *Hridayarnava Rasa*

**Fig. 5:**
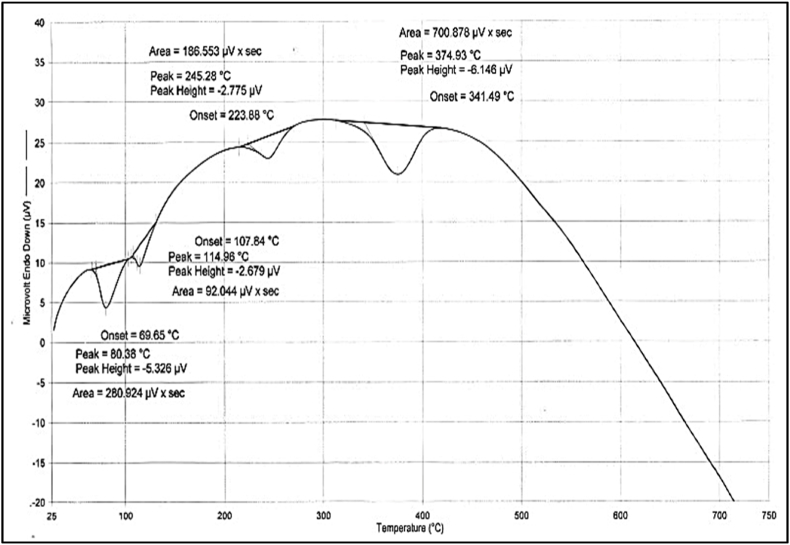
DSC analysis graph of prepared *Hridayarnava Rasa*

Thermogravimetric analysis (TGA) is the most used thermal analysis technique since it measures material reduction in weight in relation to temperature. In TGA of HR, the increasing rate of temperature was kept at 10 °C/min, and a maximum temperature of 715 °C was given. Onset temperature denotes the temperature at which the weight loss begins. Mostly peaks obtained below 400 °C indicate the presence of moisture content. If the sample contains matter in which oxidation, melting or decomposition can happen below 400 °C, then such a sample is also able to give a peak at low temperature. In this analysis, the first peak is obtained at 80.38 °C but is not accompanied by a delta Y peak, which indicates the presence of little moisture content (Table 6). Delta Y component is used to indicate strong changes in the sample mostly due to the effect of temperature on organic compounds. Decomposition of some organic molecules which get added from *Bhavana dravya* can also create a peak below 400 °C, but the absence of Delta Y below 400 °C ruled out the possibility of decomposition of organic compounds. The loss on drying value of HR is 0.92% w/w which also indicates that the first deviation is due to volatilization of moisture content. The second deviation is observed at 114.96 °C, and delta Y peak is obtained (4.71%). Some organic atoms which got impregnated during the *Shodhana* process of *Tamra* might have resulted in a second deviation. These two deviations can also represent the melting of sulphur as the melting point of sulphur is 115.21 °C. The last two peaks are accompanied by a high value of delta Y. The Shape of the curve of these two peaks is indicative of strong decomposition changes shown in Fig. 4. These changes can be attributed to the volatilization of sulphur and oxidative changes in mercury. Maximum weight loss was also observed during this phase. The results shown in Fig. 5, exhibit the combustion/oxidation of prepared HR formulation with four discrete exothermic peaks, in which the first exhibits a broad peak at 80.38 °C due to the removal of moisture content from the prepared formulation. The second DSC peak of sulphur at 114.96 °C shows its amorphous nature, as no endothermic peaks were seen. The decomposition of complex copper sulphide formed during the formulation process was noticeable by a third exothermic peak at 245.28 °C, which was found to be similar to work done by previous research groups [55,56]. The fourth DSC peak observed at 374.93 °C was found to be of an exothermic nature, indicating the thermal decomposition of complex sulphur compounds formed in the formulation process due to the reaction of elemental sulphur with copper and mercury [57]. The study results suggest that the compounds of mercury, copper and sulphur formed during the formulation process were non-toxic in nature and can be used for medicinal purposes.

**Table 6:** Thermo-gravimetric analysis (TGA) of *Hridayarnava Rasa*.

Delta Y (%)	Area (μV x sec)	Peak (°C)	Peak height (μV)	Onset (°C)
4.710	280.924	80.38	−5.326	69.65
	92.044	114.96	−2.679	107.84
23.629	186.553	245.28	−2.775	223.88
39.610	700.878	374.93	−6.146	341.49

**Fig. 6:**
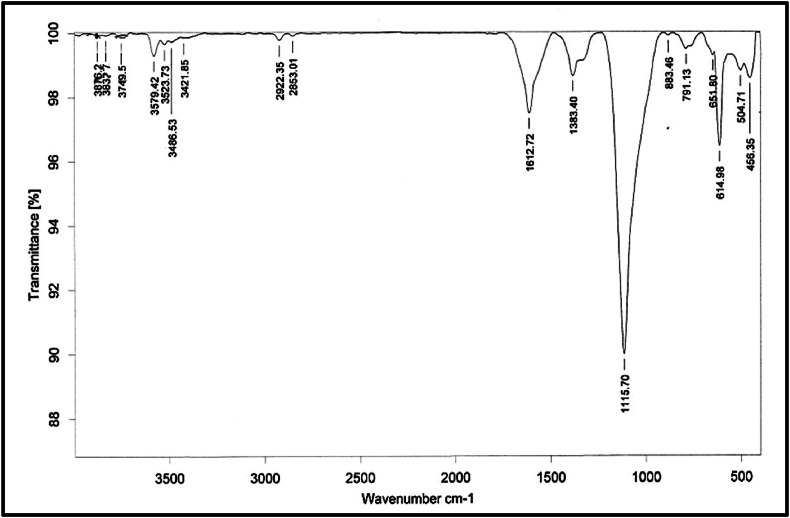
The FTIR graph of *Hridayarnava Rasa*.

An FTIR study was carried out in order to determine whether or not HR contains any functional groups or organic legends. FTIR spectra were observed in the region of 3900-450 cm^−1^, and a large number of functional groups were observed (Fig. 6). FTIR spectra are divided into four regions, as given in Table 7. An area with a frequency of less than 650 cm^−1^ that does not have a precise classification yielded four peaks. Because there was no peak found in the triple bond area, this suggests that HR does not have an excessively complicated structure. The sample's physical properties are the ones that need to be correlated with its chemical makeup in order to complete the analysis. This, in turn, can be connected to significant spectral characteristics that are present in the infrared spectrum. Colour and smell are two aspects that are very significant. Nitrogen compounds are often characterised by their related colours. The specific odor of HR is due to *Bhavana* (*Triphala Kwatha* and *Kakmachi Swarasa*) drugs, and this may result in a peak at 3421.85 cm^−1^ which is assigned to N─H stretching vibrations of aliphatic primary amine. A pattern emerges from the hydrogen bonding between atoms that mixes bands of varying sizes. These are often linked to the molecular and ionic frameworks with their symmetry as well as the complexity of the crystal architecture [58]. The peaks can be seen in the spectra at 3876.22, 3832.71, 3749.59, 3579.42, 3523.73, 3486.53, and 3421.85 cm−1, which are the stretching vibrations of the NH_2_ and –OH groups. The stretching vibrations of –CH are associated with other, less noticeable peaks at 2922.35 cm^−1^ and 2853.01 cm-1. Additionally, two separate peaks are visible at 1612.72 and 1383.40 cm^−1^, corresponding to the NH stretching and C–H alkane bending of the amide group, respectively. Finally, peaks at 1115.70 cm^−1^ indicate the stretching motions of C-O, 791.13 and 883.46 cm^−1^ show the presence of aromatic C-H of a meta-disubstituted benzene ring, 651.80 and 614.98 cm^−1^ indicate the peaks of C-Cl, 504.71 cm^−1^ indicate strong vibrations of C-Br and 456.35 cm^−1^ indicates the vibrations of C-I respectively [59,60]. The FTIR analysis strongly suggests the presence of organic groups in HR. Presence of organic matter acts as the coating material on the surface of the metallic elements and carrier of organic matter derived from herbs used during the pharmaceutical processing making the drug bio-available and thus increasing its efficacy.

**Table 7:** Various peaks obtained in FTIR analysis of *Hridayarnava Rasa* and their correlation.

No.	Peak Range	Actual peak observed	Bond	Type of bond	Specific type of bond	Appearance
1	1380 cm^−1^	1383.40	**C-H**	Alkyl	Methyl	Weak
2	2850 cm^−1^	2853.01	**C-H**	Alkyl	methylene	Medium to strong
3	2925 cm^−1^	2922.35	**C-H**	Alkyl	methylene	Medium to strong
4	750–800 cm^−1^	791.13	**C-H**	Aromatic	Meta-disub. Benzene	Strong
5	860–900 cm^−1^	883.46	**C-H**	Aromatic	Meta-disub. Benzene	Strong
6	1550–1610 cm^−1^	1612.72	**C═O**	carboxylic acids/derivates	carboxylates (salts)	
7	1550–1610 cm^−1^	1612.72	**C═O**	carboxylic acids/derivates	amino acid zwitterions	
8	3500–3560 cm^−1^	3523.73	**O-H**	carboxylic acids	low concentration	
9	3400–3500 cm^−1^	3421.85	**N─H**	primary amines	Any	
11	>3000 cm^−1^	3876.22, 3832.71, 3749.59, 3579.42, 3523.73, 3486.53, 3421.85	**N─H**	secondary amines	Any	weak to medium
12	2400–3200 cm^−1^	2922.35, 2853.01	**N─H**	ammonium ions	Any	multiple broad peaks
13	1120 cm^−1^	1115.70	**C─O**	ethers	aliphatic	
14	1100–1300 cm^−1^	1115.70	**C─O**	esters	Any	
17	540–760 cm^−1^	651.80, 614.98	**C─ Cl**	chloroalkanes	Any	weak to medium
18	500–600 cm^−1^	504.71	**C─Br**	bromoalkanes	Any	medium to strong
19	500 cm^−1^	456.35	**C─I**	iodoalkanes	Any	medium to strong
20	1380 cm^−1^	1383.40	**N─O**	nitro compounds	aliphatic	Weaker

### 4.6 PSD and CHNS-O analysis

The particle size distribution (PSD) of a powder or granular substance is a collection of numbers or a mathematical expression that describes the relative quantities of particles in existence, sorted according to size. This can also be referred to as grain size dispersal. It is essential in gaining a grasp of both the substance's physical and chemical qualities. In PSD analysis of HR, 50% material was found below 16.40 μm (Table 8).

**Table 8:** Particle size distribution (PSD) analysis of *Hridayarnava Rasa*.

Results	Particle size of *Hridayarnava Rasa* in μm
VMD	24.94
SMD	8.37
C_opt_	20.28 %
X10	2.95
X16	4.42
X50	16.40
X84	48.44
X90	59.65
X99	103.32

SMD is for standard mean diameter; VMD stands for volumetric mean diameter; C_opt_ stands for optimal concentration; ×10: Ten percent of the material is smaller than the micron figure that was given; ×16 indicates that 16% of the material is smaller than the value mentioned in the micron range; ×50 indicates that 50% of the material is smaller than the value mentioned in the micron range; ×84 indicates that 84% of the material is smaller than the value mentioned in the micron range; ×90 indicates that 90% of the material is smaller than the value mentioned in the micron range; and ×99 indicates that 99% of the material is smaller than the value mentioned in the micron range.

In present study, ingredients of HR were triturated for around 10 h in porcelain mortar along with the *Triphala Kwatha* and *Kakmachi Swarasa*. During the wet trituration procedure, the material is broken down into small particles due to the application of force in the form of rubbing/trituration/friction between mortar and pestle. It is also called attrition. Attrition or milling is an example of a common “top-down approach” to the production of nanoparticulate structures. This method includes the fragmentation of huge amounts of material in order to produce the necessary nanostructures from those big bits of material. The process of preparation of Ayurvedic Herbo-mineral-metallic formulations involves steps like *Shodhana*, *Marana*, *Bhavana* etc, which are the ancient methods of reducing the particle size up to the nano level. Recent microstructural analyses of several metallic *Bhasma* have confirmed their status as nanoparticles. The surface area and surface energy of particles grow by a factor of seven as they shrink from centimetres to nanometres. These particles are thermodynamically unstable or metastable since their large surface area gives them a high surface energy [61,62]. Herbo-mineral-metallic compositions may have a longer shelf life because of their metastability (long-lived but not genuinely indefinite stability) [63]. A faster dissolving rate and easier absorption of a medicine result from smaller particles and a larger surface area. The drug's bioavailability is therefore, significantly improved. These variables (increased potency, smaller particle size, etc.) allow for lower doses of *Bhasma*, which in turn reduces dose-related adverse effects. This may be the reason why *Bhasma* and Herbo-mineral-metallics formulations work so quickly and are so effective, even in smaller doses.

### 4.7 CHNS-O analysis of *Hridayarnava Rasa*

Since the sulphur is an ingredient of *Samaguna Kajjali* and *Tamra Bhasma*, the elemental percentage of sulphur was found to be significantly high in this analysis also (Table 9). Carbon in the finished drug sample shows the presence of organic materials which are impregnated during the procedure of wet trituration (*Bhavana*).

**Table 9:** CHNS-O analysis of *Hridayarnava Rasa*.

Peak No.	Component	Retention time (min)	Area	Element %
1	Nitrogen	0.817	57,531	0.983
2	Carbon	1.208	436,692	3.318
3	Hydrogen	3.167	344,837	0.889
4	Sulphur	6.050	1,111,410	22.385
5	Oxygen	0.433	677,041	6.111

## 5 Conclusion

The results of current study performed showed the successful design and formulation of an Ayurvedic Herbo-mineral-metallic formulation *Hridayarnava Rasa* and its evaluation by validated standard analytical procedures mentioned in classical ayurvedic texts, Ayurvedic Pharmacopoeia of India, and advanced instrumentation techniques. This study will be helpful in the establishment of quality standards for the preparation of *Hridayarnava Rasa* to produce a product of high quality, high efficacy, and safety with minimal side effects. The *Bhavana* process has been believed to make the *ras-aushadhis* non-hazardous and safer to be administered without having adverse drug reactions [64]. Therefore, based on the results of the study worth of work, we can state that particle size, elements like carbon, hydrogen, oxygen, nitrogen, and sulphur, advanced analytical technique like CHNSO analyzer, ICP-AES, EDAX, crystalline structure by XRD, particle morphology by SEM, FTIR, TGA-DSC will be helpful to determine molecular interaction in HR with other ingredients. The Herbo-mineral-metallic formulation that was prepared in the present study and employed various advanced analytical techniques to analyze it is what made it possible for us to successfully analyses complicated multicomponent compounds that are present in it. The clinical studies are further needed to be explored for the efficacy of such herbo-mineral-metallic formulations.

## Sources of funding

This work was funded by Institute for Post Graduate Teaching and Research in Ayurveda, Gujarat Ayurved University, Jamnagar.

## Author contributions

Chandrashekhar Y. Jagtap: Conceptualization, Writing – original draft, Study design. Ashwini Kumar Mishra: Writing – original draft, review & editing, Formal analysis, Software. Mukesh Nariya: Supervision, Project administration, Investigation. Vinay J. Shukla: Visualization, Study design, Formal analysis. Pradeep Kumar Prajapati: Supervision, Funding acquisition, Project administration.

## Declaration on use of generative AI in scientific writing

The authors declare that they have not used any generative AI tool or AI assisted technologies while writing this manuscript.

## Conflict of interest

The authors announce that there are no competing interests.
