# Supplementary material for: Product development and characterization of the Ayurvedic herbo-mineral-metallic compound- Hridayarnava Rasa
Source: J Ayurveda Integr Med. 2024 May 17;15(3):100886. doi: 10.1016/j.jaim.2024.100886 (PMC11127204; doi:10.1016/j.jaim.2024.100886)
Supplement: Multimedia component 1 [file mmc1.docx]

**Supplementary Data**

**Supplementary Table 1: Percentage of copper and other elements in raw copper and after *Samanya* and *Vishesha* *Shodhana* of *Tamra***

| **Parameters** | **Raw *Tamra*** | **After *Samanya Shodhana*** | **After *Vishesha Shodhana*** |
| --- | --- | --- | --- |
| Copper (%) | 99.800 | 89.76 | 88.10 |
| Iron (%) | 0.029 | 8.87 | 7.62 |
| Zinc (%) | 0.032 | 0.021 | 0.022 |
| Lead (%) | 0.032 | 0.029 | 0.030 |
| Tin (%) | 0.001 | 0.001 | 0.001 |
| Silver (%) | 0.051 | 0.046 | 0.045 |
| Arsenic (%) | ˂0.0004 | ˂0.0002 | ˂0.0002 |
| Cadmium (%) | ˂0.0009 | ˂0.0008 | ˂0.0008 |
| Aluminum (%) | 0.011 | 0.001 | 0.010 |
| Sulphur (%) | 0.003 | 0.002 | 0.002 |

**Supplementary Table 2: Percentage of sulfur and mercury in raw and *Shuddha* samples of *Gandhaka* and *Parada***

| **Parameter** | **Raw *Gandhaka*** | ***Shuddha Gandhaka*** | **Raw *Parada*** | ***Shuddha Parada*** |
| --- | --- | --- | --- | --- |
| Sulfur (%w/w) | 96.84 | 93.48 | **--** | **--** |
| Mercury (%w/w) | **--** | **--** | 98.56 | 90.67 |

**Supplementary Table 3:** **Analysis of liquid media before and after *Samanya* *Shodhana* of *Tamra***

| **Material** | **Parameter** | **Before** | **After** |
| --- | --- | --- | --- |
| *Tila Taila* | Specific gravity | 0.9242 | 0.9253 |
|  | Refractive index | 1.48 | 1.48 |
| *Takra* | pH | 4.33 | 4.69 |
|  | Total solid content (%w/v) | 5.72 | 6.34 |
|  | Specific gravity | 1.021 | 1.41 |
| *Gomutra* | pH | 7.51 | 7.58 |
|  | Total solid content (%w/v) | 6.034 | 7.17 |
|  | Specific gravity | 1.038 | 1.042 |
| *Kanji* | pH | 2.31 | 2.41 |
|  | Total solid content (%w/v) | 5.167 | 5.504 |
|  | Specific gravity | 1.502 | 1.066 |
| *Kulattha Kwatha* | pH | 7.74 | 7.38 |
|  | Total solid content (%w/v) | 8.427 | 8.514 |
|  | Specific gravity | 1.036 | 1.039 |

**Supplementary Table 4: Classical chemical parameters of *Tamra* *Bhasma* prepared from *Shuddha* *Tamra***

| **Parameters** | ***Tamra* *Bhasma*** |
| --- | --- |
| *Rekhapurnatwa* | Test Passed |
| *Varitaratwa* | Test Passed |
| *Sukshmatwa* | Test Passed |
| *Nischandratwa* | Test Passed |
| *Mridutwa* | Test Passed |
| *Niswadutwa* | Test Passed |
| *Dadhi Pariksha* | Test Passed |
| *Niruttha* | Test Passed |
| *Apunarbhavatwa* | Test Passed |
